# Contributory role of SARS-CoV-2 genomic variations and life expectancy in COVID-19 transmission and low fatality rate in Africa

**DOI:** 10.1186/s43042-020-00116-x

**Published:** 2020-12-09

**Authors:** Olabode E. Omotoso

**Affiliations:** Cancer Research and Molecular Biology Laboratories, University of Ibadan, Ibadan, Nigeria

**Keywords:** Coronavirus, Mutations, Low fatality, Age, SARS-CoV-2, Africa

## Abstract

**Background:**

The novel coronavirus disease (COVID-19) has claimed lots of lives, posing a dire threat to global health. It was predicted that the coronavirus outbreak in the African population would be very lethal and result to economic devastation owing to the prevalence of immune-compromised population, poverty, low lifespan, fragile health care systems, poor economy, and lifestyle factors. Accumulation of mutations gives virus selective advantage for host invasion and adaptation, higher transmissibility of more virulent strains, and drug resistance. The present study determined the severe acute respiratory syndrome-2 (SARS-CoV-2) genomic variability and the contributory factors to the low COVID-19 fatality in Africa. To assess the SARS-CoV-2 mutational landscape, 924 viral sequences from the Africa region with their sociobiological characteristics mined from the Global Initiative on Sharing All Influenza Data (GISAID) database were analyzed.

**Results:**

Mutational analysis of the SARS-CoV-2 sequences revealed highly recurrent mutations in the SARS-CoV-2 spike glycoprotein D614G (97.2%), concurrent R203K, and G204R (65.2%) in the nucleocapsid phosphoprotein, and P4715L (97.2%) in the RNA-dependent RNA polymerase flagging these regions as SARS-CoV-2 mutational hotspots in the African population. COVID-19 is more severe in older people (> 65 years); Africa has a low percentage of people within this age group (4.36%). The average age of the infected patients observed in this study is 46 years with only 47 infected patients (5.1%) above 65 years in Africa in comparison to 13.12% in countries in other continents with the highest prevalence of COVID-19.

**Conclusions:**

Africa’s young generation, the late incidence of the disease, and adherence to public health guidelines are important indicators that may have contributed to the observed low COVID-19 deaths in Africa. However, with the easing of lockdown and regulatory policies, daily increasing incidence in most countries, and low testing and sequencing rate, the epidemiology and the true impact of the pandemic in Africa remain to be unraveled.

## Background

Severe acute respiratory syndrome coronavirus 2 (SARS-CoV-2), the causative pathogen of the novel coronavirus disease 2019 (COVID-19), was first reported in December 2019 in Wuhan, China [1, 2]. The highly infectious SARS-CoV-2 has spread globally within 3-months of its first outbreak and has posed dire stress on public health and global economy [1, 3]. Globally, as of October 16, 2020, there have been 39,153,270 confirmed cases and 1,102,440 reported deaths due to COVID-19 [4]. It was predicted that the coronavirus outbreak in the African population would be very lethal and result to economic devastation owing to the prevalence of immune-compromised population (due to HIV/AIDS, tuberculosis, hepatitis virus, malaria, etc.), poverty, low lifespan, fragile health care systems, poor economic decisions, and lifestyle factors [3, 5]. Africa was the last continent to be hit, and surprisingly boast of about 80% recovery rate at this stage of the pandemic [6]. Africa accounts for 3.5% of the fatality rate recorded globally with the highest death toll reported in Americas (55.1%) and Europe (25.7%) which are developed regions with high standard of living and healthcare structure [6, 7]. In line with public health and the World Health Organization (WHO) guidelines, the African governments and policy makers were quick to institute policies (restrictions such as partial/full-scale lockdowns, physical distancing, accelerated contact tracing, self-isolation, and quarantine) and adopt public health measures in light of the index case in the continent [7–9]. Africa’s index case of COVID-19 was on the 14th of February, 2020, in Egypt, North Africa [10] and on the 27th of February, Nigeria; Sub-Saharan Africa reported its index case from a European traveler [11]. As of October 16, 2020, there have been 1,623,181 confirmed cases and 39,150 deaths in Africa, out of which South Africa has the highest toll with 698,184 confirmed cases and 18,309 deaths [4].

Coronaviruses are single-positive-stranded RNA viruses with the largest genome (ranging from 26 to 32 kb) among all RNA viruses consisting of *ORF1ab* (21,289 nucleotides), *ORF3a* (827 nucleotides), *ORF3b* (455 nucleotides), *ORF6* (185 nucleotides), *ORF7a* (365 nucleotides), *ORF7b* (131 nucleotides), *ORF8* (365 nucleotides), *ORF10* (116 nucleotides), and four genes encoding structural proteins: the *nucleocapsid* (1259 nucleotides), *membrane* (668 nucleotides), *envelope* (227 nucleotides), and *spike* (3,821 nucleotides) [1, 12–14]. Accumulation of mutation is central to viral evolution, transmission, and virulence. This gives virus selective advantage for host invasion and adaptation, higher transmissibility of more virulent strains, and drug resistance [9, 14, 15]. Identifying the mutational pattern of coronaviruses and monitoring their spread can be important in guiding drug/vaccine development and in making decisions to curtail the transmission [16]. The present study aims to gain insight into the mutational landscape of the SARS-CoV-2 genome in African population which may serve as eventual targets for drug design and/or vaccine development. This study also assesses the contributory factors to the low fatality rate due to COVID-19 in the African continent.

## Methods

### Data acquisition

A slightly modified protocol was used [17]. A total of 924 SARS-CoV-2 genomic sequences from the Global Initiative on Sharing All Influenza Data (GISAID) database (https://www.epicov.org/epi3/frontend) filtered as “high coverage only, *Homo sapiens*, complete, all clades and low coverage excl”, with patient’s status, “Africa” were mined. Patient’s age of all the sequences were also obtained to determine the age distribution of the infected patients. The accession numbers and laboratories in Africa that sequenced and deposited complete SARS-CoV-2 genomes on the GISAID database used in this study are provided as Supplementary file S1.

Country data of number of tests done per country with each nation’s population, confirmed cases, recoveries, and reported deaths due to COVID-19 were obtained from Worldometer (https://www.worldometers.info/coronavirus/) and the WHO database (covid19.who.int). The age distribution of countries with highest prevalence of COVID-19 cases was obtained from the World Factbook (www.cia.gov).

### Sequence and mutational analysis

In this study, the mined 924 SARS-CoV-2 viral sequences were used to analyze the genomic variability since the index case of COVID-19 pandemic in Africa in February 2020 to identify the frequency and spread of mutations in the African population. The evolution of COVID-19 outbreak with respect to the transmission in the mutational hotspots was assessed and evaluated on the GISAID web interface (https://www.epicov.org/epi3/frontend). Recurrent mutations observed were focused on as they are likely to confer viral-host structure-function relationship promoting higher transmission rate.

### Determination of testing, fatality, and recovery rate

The testing rate was determined for each African country as percentage of total test done from the country’s population.

$$ \mathrm{Testingrate}\left(\%\right)=\frac{\mathrm{TotalCOVID}-19\mathrm{testdone}}{\mathrm{Countr}{\mathrm{y}}^{\prime}\mathrm{spopulation}}\times 100 $$

The fatality rate was determined as percentage of total reported deaths due to COVID-19 from each country’s number of confirmed cases.

$$ \mathrm{Fatality}\ \mathrm{rate}\ \left(\%\right)=\frac{\mathrm{Total}\ \mathrm{number}\ \mathrm{of}\ \mathrm{COVID}-19\ \mathrm{reported}\ \mathrm{deaths}\ }{\mathrm{Number}\ \mathrm{of}\ \mathrm{confirmed}\ \mathrm{cases}}\times 100 $$

The recovery rate was determined as percentage of number of infectious patients who recovered from all reported confirmed cases in each country.

$$ \mathrm{Recovery}\ \mathrm{rate}\ \left(\%\right)=\frac{\mathrm{Number}\ \mathrm{of}\ \mathrm{COVID}-19\ \mathrm{infected}\ \mathrm{patients}\ \mathrm{who}\ \mathrm{recovered}}{\mathrm{Number}\ \mathrm{of}\ \mathrm{confirmed}\ \mathrm{cases}}\times 100 $$

## Results

Genomic variabilities dispersed at various sites in the SARS-CoV-2 sequence were observed; few mutations occurred more frequently (presented in Table 1). Mutational analysis of the 924 SARS-CoV-2 sequences revealed highly recurrent mutations in D614G (898 viral sequences) which falls in the spike glycoprotein (S) region and was observed concurrently with P4715L variant (898 viral sequences) in the ORF1ab polyprotein region. More so, R203K and G204R mutations (602 viral sequences respectively) in the nucleocapsid (N) phosphoprotein region, Q57H (60 viral sequences) in the ORF3a region, L84S (16 viral sequences) in the ORF8 region, and L3606F (45 viral sequences), G3278S (73 viral sequences), and T265I (26 viral sequences) in the ORF1ab polyprotein region were observed. It is worthy of note that 97.3% of viral sequences with the nsp5 G3278S mutation were from South Africa. The distribution of the mutations flags the Spike S1 domain (D614G), N protein R203K and G204R and nsp12 P4715L as SARS-CoV-2 mutational hotspots in the African population. According to mutational pattern, the viral sequences were characterized into the GISAID six (6) clades: GR (598), G (240), GH (57), S (16), O (7), V (4), and L (2).

**Table 1:** Recurrent mutations observed in the viral samples (*N* = 924 viral sequences)

S/N	SARS-CoV-2 region	Mutation observed	Occurrence
1	Spike protein; S1 domain	L18F	2
2		A222V	4
3		D614G	898
4	Spike protein; S2 domain	E780Q	2
5	Nucleocapsid phosphoprotein	P13L	2
6		R203K	602
7		G204R	602
8	ORF3 protein	Q57H	60
9		G251V	4
10	ORF8 protein	L84S	16
11	ORF1ab polyprotein; nsp2	T265I	26
12		D448del^a^	6
13		I739V	3
14		P765S	3
15	ORF1ab polyprotein; nsp5	G3278S	73
16	ORF1ab polyprotein; nsp6	L3606F	45
17	ORF1ab polyprotein; RdRp	A4489V	3
18		P4715L	898

Highly recurrent gene variants were observed in the *spike*, *nucleocapsid*, and *ORF1ab*, flagging these genome segments as SARS-CoV-2 mutational hotspots

^a^Key: *RdRp* RNA dependent RNA polymerase. One letter code for corresponding amino acid: *A* alanine, *D* aspartic acid, *G* glycine, *E* glutamate, *R* arginine, *K* lysine, *Q* glutamine, *H* histidine, *V* valine, *L* leucine, *S* serine, *T* threonine, *I* isoleucine, *P* proline, and *F* phenylalanine

**Fig. 1:**
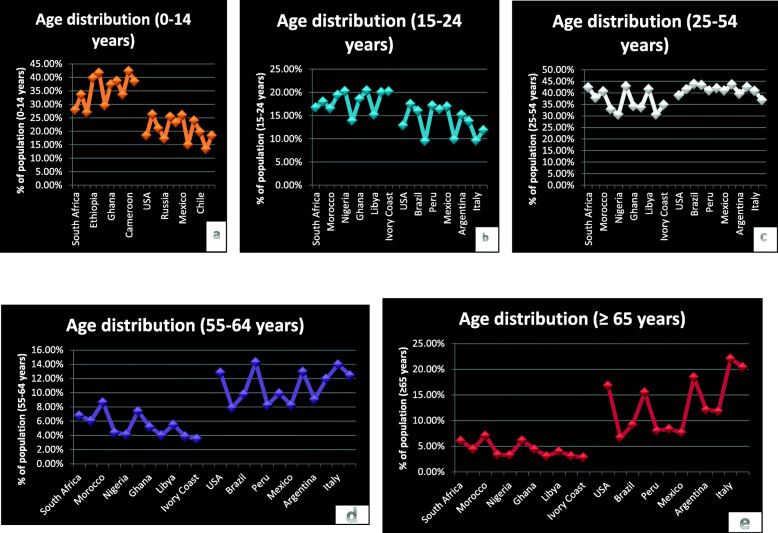
**a**–**e** Age distribution of African countries in comparison with nations from other continents with the highest prevalence of COVID-19. This reveals a high prevalence of younger generations in Africa (0–14 years), with equilibrium at age range 15–54 years when compared to older population (≥ 55 years) in other regions studied with high COVID-19 prevalence

For the age distribution, the average age of the infected patients is 46 years with only 47 infected patients (5.1%) above 65 years. According to demographic data from the World Factbook, the percentage of population of Africans ≥ 65 years is 4.36% in comparison to 13.12% in countries in other continents with highest prevalence of COVID-19 (as shown in Fig. 1a–e). At this stage of the pandemic, Africa still faces the challenge of low testing as none of the African countries meet up with the 15% testing rate standard, as South Africa has the highest testing rate (6.59%) in Africa. Nevertheless, there is a high recovery rate and low fatality rate observed across the African continent. The trend and distribution of confirmed cases, discharged cases, fatality, and recovery rate are as shown in Table 2.

**Table 2:** Trend and distribution of confirmed cases, discharged cases, and fatality rate across Africa

S/No	Country	Confirmed cases	Total deaths	Recovered cases	Total test done	Population	Testing rate	Fatality rate	Recovery rate
	Africa	1,359,569	32,678	1,104,047				2.40%	81.21%
1	South Africa	649,793	15,447	577,906	3,918,478	59,461,240	6.59%	2.38%	88.94%
2	Egypt	101,009	5648	84,161	135,000	102,722,504	0.13%	5.59%	83.32%
3	Morocco	86,686	1,578	67,528	2,230,069	36,999,965	6.03%	1.82%	77.90%
4	Ethiopia	64,301	1013	24,983	1,138,012	115,528,092	0.99%	1.58%	38.85%
5	Nigeria	56,256	1082	44,152	440,248	207,152,368	0.21%	1.92%	78.48%
6	Algeria	48,254	1,612	34,037		44,010,565		3.34%	70.54%
7	Ghana	45,434	286	44,342	450,872	31,202,737	1.44%	0.63%	97.60%
8	Kenya	36,157	622	23,067	481,982	54,007,662	0.89%	1.72%	63.80%
9	Libya	22,781	362	12,183	157,481	6,890,311	2.29%	1.59%	53.48%
10	Cameroon	20,167	415	18,837	149,000	26,676,814	0.56%	2.06%	93.41%
11	Ivory Coast	19,013	120	18,112	142,248	26,507,763	0.54%	0.63%	95.26%
12	Madagascar	15,757	211	14,368	64,174	27,831,811	0.23%	1.34%	91.18%
13	Senegal	14,280	297	10,520	158,751	16,831,055	0.94%	2.08%	73.67%
14	Zambia	13,539	312	12,260	131,034	18,485,203	0.71%	2.30%	90.55%
15	Sudan	13,516	835	6757		44,052,920		6.18%	49.99%
16	DRC	10,390	264	9756		90,093,630		2.54%	93.90%
17	Guinea	10,045	63	9292	28,140	13,203,001	0.21%	0.63%	92.50%
18	Namibia	9719	101	6543	80,352	2,550,174	3.15%	1.04%	67.32%
19	Gabon	8643	53	7706	135,615	2,236,175	6.06%	0.61%	89.16%
20	Zimbabwe	7526	224	5678	154,733	14,906,868	1.04%	2.98%	75.45%

This shows the countries with highest prevalence of COVID-19 in Africa, the data for all African countries is available as Suppl. file S2. The fatality due to COVID-19 in Africa is 2.4% with 81.21% recovery rate. South Africa has the highest COVID-19 testing rate (about 7%) in Africa

## Discussion

As of September 5, 2020, over 705 million people in the world live in extreme poverty compared to about 651 million people in 2019 [5]. In 2020, 102,125,917 people are reported to be living in extreme poverty from a total population of 205,323,504 people in Nigeria, the most populous African nation compared to 200,369,248 people, among whom 92,441,550 people lived in extreme poverty in 2019 [5]. The COVID-19 has significantly ravaged economies and posed serious threat to public health and social interaction, spreading across the globe within 3 months of its outbreak [3]. It was hypothesized that low- and middle-income countries, especially African nations, would be seriously hit by the pandemic due to her vulnerability to infectious diseases and inadequate health structure. Surprisingly, there have been very low mortality rate due to COVID-19 in African nations when compared to developed nations in the Americas and Europe where the pandemic has had its highest toll of infection and fatality [7]. This study assessed the possible factors that could have accounted for the low mortality rate due to COVID-19 in Africa.

The dataset from infected patients helps to understand the COVID-19 infection rate, risks, epidemiology, and spread of the disease. The WHO suggested 10–30 tests per confirmed case as an indicator of adequate testing [7]. South Africa has the highest test rate of 3,918,478 people in Africa, representing about 7% of its 59,461,240 population, which is still a way long off from the adequate testing indicator and also when compared with countries with highest prevalence of COVID-19 in other regions: USA (37%), Russia (36%), and Australia (32%) [4]. In same vein, Nigeria, the most populous Black African nation, has only tested 440,248 people representing 0.21% of its over 207 million population. This trend holds true for many other African countries. Hence, caution should be taken while interpreting these data as the true number of COVID-19 cases in Africa might still remain undetected. However, with the current data available, though with daily increasing incidence, the mortality rate due to COVID-19 in Africa has been minimal. Other factor such as late incidence of the disease [10, 18], which gives an advantage for early preparation before the outbreak by enforcing regulatory guidelines (active surveillance, isolation, quarantine, contact tracing, and social distancing among others) in line with the WHO guidelines could have played a key role in managing the pandemic in Africa [7, 8]. The American Public Media (APM) Research laboratory in August 2020 reported 88.4 deaths per 100,000 Black Americans due to COVID-19 compared to 54.4 deaths per 100,000 Latino Americans and 36.4 deaths per 100,000 Asian Americans. The APM findings suggests higher mortality due to COVID-19 in Black Americans in the USA [19]. This raises a concern to understand why Blacks are dying more due to COVID-19 overseas than in Africa.

The present study also looked into the mutational landscape of SARS-CoV-2 genome in Africa. Highly recurrent mutation (D614G) was observed in the S1 domain of S glycoprotein which facilitates viral entry into host by binding to the human angiotensin-converting enzyme 2 (ACE2) receptor. The S2 domain of the S glycoprotein which plays a crucial role in viral-host cell membrane fusion was well conserved; this could serve as an important target for antiviral drug design. The T265I mutation that is prevalent in the USA which aids SARS-CoV-2 survival in the host cell [20] was only observed in 26 viral sequences, predominantly in Senegal (69.23%). P4715L mutation hotspot present in the RNA-dependent RNA polymerase (RdRp) region was first observed in Italy during the sporadic increase in incidence and fatality of COVID-19 in Europe [15]. This supports the evidence that most of Africa’s index case originates from European and American travelers [3, 18], which could have resulted to the vast distribution of these mutations across the infected patients.

The concurrent R203K and G204R mutation are present in the N phosphoprotein region, which plays an important role in viral interaction with the M protein during virion assemblage [21]. Gene variants in the *nucleocapsid* region alter miRNAs binding, which might contribute to the pathogenesis and progression of infection in the patient [22]. The viral ORF3a and ORF10 proteins can synergistically attack heme on the host’s hemoglobin 1-β chain, thereby disintegrating iron to form porphyrin. This results to reduced hemoglobin-carrying oxygen and carbon dioxide causing extreme poisoning and inflammation of the hepatocytes [23]. Q57H mutation in the ORF3a region was observed to coincide with D614G and P4715L variants. The RdRp P4715L mutation coincides with S protein D614G mutation alongside the concurrent N protein R203K and G204R variants, and in their absence ORF3a Q57H variant. The SARS-CoV-2 membrane, envelope, ORF6, ORF7, and ORF10 proteins were well conserved. These mutational hotspots and conserved domains must be well-considered during drug or vaccine design to avoid vaccine evasion and drug resistance.

Lastly, this study looked at the age distribution of the infected patients as a contributory factor to the low fatality rate. The average life expectancy in Nigeria is 54 years, a reflection of Sub-Saharan Africa (61 years) compared to European union (81 years), China (77 years), and USA (79 years) [24]. Earlier report [3] suggests that COVID-19 has high mortality and severity in older people (> 65 years) than the younger population, which have high chances of recovery from the infection. Only 5.1% of the 924 infected Africans in this present study are over 65 years. This could have played a major role in the relatively high recovery rate (81.21%) and low mortality (2.4%) due to COVID-19 observed in the African population.

## Conclusion

As of now, the highly infectious COVID-19 continues to spread globally, with increasing incidence and mortality. The African continent continues to enjoy the benefit of the swift actions and policies imposed to curtail the pandemic. With the gradual easing of these measures, increasing poverty, poor healthcare structure, and a large percentage of immune-compromised population, it is important that the populace adhere to regulatory guidelines. Low life expectancy, low testing rate, late incidence of the disease, and adherence to public health guidelines could be important factors that have contributed to the observed low COVID-19 fatality in Africa. However, sufficient data is still unavailable to ascertain the epidemiology, transmission, genomic variation, and the true impact of the pandemic in Africa. Genomic sequences are important in the design and development of antiviral drugs and vaccine. From the over 200,000 complete SARS-CoV-2 sequences available in public repositories, Africa has barely contributed 4000 viral sequences (2%). Collaboration with scientists and research institutes in African nations is highly recommended so as to enhance their delivery capacity.

## Supplementary Information

**Additional file 1:** Supplementary file S1R1.

**Additional file 2:** Supplementary file S2R1.

## Data Availability

All data used in this study are available in recognized public repositories as indicated.

## Abbreviations

ACE2: Angiotensin-converting enzyme 2
APM: American Public Media
COVID-19: Coronavirus disease 2019
E: Envelope protein
GISAID: Global Initiative on Sharing All Influenza Data
M: Membrane protein
N: Nucleocapsid phosphoprotein
RdRp: RNA-dependent RNA polymerase
S: Spike glycoprotein
SARS-CoV-2: Severe acute respiratory syndrome coronavirus 2
WHO: World Health Organization
