# Supplementary material for: Contributory role of SARS-CoV-2 genomic variations and life expectancy in COVID-19 transmission and low fatality rate in Africa
Source: Egypt J Med Hum Genet. 2020 Dec 9;21(1):72. doi: 10.1186/s43042-020-00116-x (PMC7723464; doi:10.1186/s43042-020-00116-x)
Supplement: Supplementary file 2 — Additional file 2: Supplementary file S2R1. [file 43042_2020_116_MOESM2_ESM.pdf]

S/No	Country	Confirmed cases	Total deaths	Recovered cases	Total test done	Population	Testing rate	Fatality rate	Recovery rate
	Africa	1,359,569	32,678	1,104,047				2.40%	81.21%
1	South Africa	649,793	15,447	577,906	3,918,478	59,461,240	6.59%	2.38%	88.94%
2	Egypt	101,009	5,648	84,161	135,000	102,722,504	0.13%	5.59%	83.32%
3	Morocco	86,686	1,578	67,528	2,230,069	36,999,965	6.03%	1.82%	77.90%
4	Ethiopia	64,301	1,013	24,983	1,138,012	115,528,092	0.99%	1.58%	38.85%
5	Nigeria	56,256	1,082	44,152	440,248	207,152,368	0.21%	1.92%	78.48%
6	Algeria	48,254	1,612	34,037		44,010,565		3.34%	70.54%
7	Ghana	45,434	286	44,342	450,872	31,202,737	1.44%	0.63%	97.60%
8	Kenya	36,157	622	23,067	481,982	54,007,662	0.89%	1.72%	63.80%
9	Libya	22,781	362	12,183	157,481	6,890,311	2.29%	1.59%	53.48%
10	Cameroon	20,167	415	18,837	149,000	26,676,814	0.56%	2.06%	93.41%
11	Ivory Coast	19,013	120	18,112	142,248	26,507,763	0.54%	0.63%	95.26%
12	Madagascar	15,757	211	14,368	64,174	27,831,811	0.23%	1.34%	91.18%
13	Senegal	14,280	297	10,520	158,751	16,831,055	0.94%	2.08%	73.67%
14	Zambia	13,539	312	12,260	131,034	18,485,203	0.71%	2.30%	90.55%
15	Sudan	13,516	835	6,757		44,052,920		6.18%	49.99%
16	DRC	10,390	264	9,756		90,093,630		2.54%	93.90%
17	Guinea	10,045	63	9,292	28,140	13,203,001	0.21%	0.63%	92.50%
18	Namibia	9,719	101	6,543	80,352	2,550,174	3.15%	1.04%	67.32%
19	Gabon	8,643	53	7,706	135,615	2,236,175	6.06%	0.61%	89.16%
20	Zimbabwe	7,526	224	5,678	154,733	14,906,868	1.04%	2.98%	75.45%
21	Mauritania	7,276	161	6,825	76,256	4,673,795	1.63%	2.21%	93.80%
22	Tunisia	6,635	107	1,991	171,786	11,843,970	1.45%	1.61%	30.01%
23	Malawi	5,690	177	3,731	48,556	19,227,686	0.25%	3.11%	65.57%
24	Djibouti	5,395	61	5,330	71,757	990,918	7.24%	1.13%	98.80%

25	Mozambique	5,269	35	2,960	114,333	31,427,632	0.36%	0.66%	56.18%
26	Eswatini	5,075	98	4,229	31,061	1,162,627	2.67%	1.93%	83.33%
27	Equatorial Guinea	4,996	83	4,490	55,783	1,411,938	3.95%	1.66%	89.87%
28	Congo	4,928	88	3,887		5,545,022		1.79%	78.88%
29	Cabo Verde	4,813	44	4,119	76,576	557,224	13.74%	0.91%	85.58%
30	Uganda	4,799	55	2,256	423,214	46,022,522	0.92%	1.15%	47.01%
31	CAR	4,749	62	1,825	31,459	4,846,711	0.65%	1.31%	38.43%
32	Rwanda	4,591	22	2,556	462,896	13,015,862	3.56%	0.48%	55.67%
33	Gambia	3,405	103	1,723	14,410	2,430,016	0.59%	3.02%	50.60%
34	Somalia	3,389	98	2,803		15,980,442		2.89%	82.71%
35	Angola	3,388	134	1,301	64,747	33,065,753	0.20%	3.96%	38.40%
36	Mayotte	3,374	40	2,964	13,000	274,122	4.74%	1.19%	87.85%
37	Mali	2,924	128	2,285	44,439	20,365,308	0.22%	4.38%	78.15%
38	Réunion	2,805	15	1,313	35,419	896,631	3.95%	0.53%	46.81%
39	South Sudan	2,578	49	1,290	12,044	11,220,552	0.11%	1.90%	50.04%
40	Guinea-Bissau	2,275	39	1,127		1,977,247		1.71%	49.54%
41	Benin	2,267	40	1,942	158,881	12,185,927	1.30%	1.76%	85.66%
42	Botswana	2,252	10	546	138,290	2,361,144	5.86%	0.44%	24.25%
43	Sierra Leone	2,109	72	1,636		8,009,487		3.41%	77.57%

44	Burkina Faso	1,707	56	1,135		21,016,156		3.28%	66.49%
45	Togo	1,572	37	1,190	77,212	8,317,313	0.93%	2.35%	75.70%
46	Liberia	1,319	82	1,210		5,081,319		6.22%	91.74%
47	Lesotho	1,245	33	528	15,632	2,145,750	0.73%	2.65%	42.41%
48	Niger	1,180	69	1,104	9,052	24,375,373	0.04%	5.85%	93.56%
49	Chad	1,084	80	938		16,518,393		7.38%	86.53%
50	Sao Tome and Principe	906	15	870	4,310	219,978	1.96%	1.66%	96.03%
51	Tanzania	509	21	183		60,068,408		4.13%	35.95%
52	Burundi	472	1	374	27,836	11,960,116	0.23%	0.21%	79.24%
53	Comoros	456	7	415		873,304	0.00%	1.54%	91.01%
54	Mauritius	361	10	335	227,409	1,272,209	17.88%	2.77%	92.80%
55	Eritrea	361		304		3,556,406		0.00%	84.21%
56	Seychelles	139		136	5,200	98,473	5.28%	0.00%	97.84%
57	Western Sahara	10	1	8		600252		10.00%	80.00%
	Total:	1,359,569	32,678	1,104,047				2.40%	81.21%
